# Adjuvant Epigenetic Therapy of Decitabine and Suberoylanilide Hydroxamic Acid Exerts Anti-Neoplastic Effects in Acute Myeloid Leukemia Cells

**DOI:** 10.3390/cells8121480

**Published:** 2019-11-21

**Authors:** Sonia Abou Najem, Ghada Khawaja, Mohammad Hassan Hodroj, Patil Babikian, Sandra Rizk

**Affiliations:** 1 Department of Biological Sciences, Faculty of Science, Beirut Arab University, Debbieh 5664, Lebanon; Sonia.abounajem@lau.edu.lb (S.A.N.); ghada.khawaja@bau.edu.lb (G.K.); 2 Department of Natural Sciences, School of Arts and Sciences, Lebanese American University, Beirut 1102-2801, Lebanon; mohammadhassan.hodroj@lau.edu (M.H.H.); patil.babikian@lau.edu (P.B.)

**Keywords:** epigenetics, acute myeloid leukemia, decitabine, suberoylanilide hydroxamic acid

## Abstract

Atypical epigenetic processes including histone acetylation and DNA methylation have been identified as a fundamental theme in hematologic malignancies. Such mechanisms modify gene expression and prompt, in part at least, the initiation and progression of several malignancies including acute myeloid leukemia. In the current study we determined the effects of treating KG-1 and U937 acute myeloid leukemia (AML) cells, in vitro, with the HDAC inhibitor, suberoylanilide hydroxamic acid (SAHA), or with a DNMT inhibitor, decitabine (DAC), or their combination, on cell proliferation, cell cycle progression, apoptosis, and expression of apoptosis-related proteins. Each of SAHA and DAC attenuated cell proliferation and induced cell cycle arrest and apoptotic cell death of KG-1 and U937 cell lines. Besides, their sequential combination improved the obtained anti-neoplastic effect: significant augmentation of growth inhibition and apoptosis induction as compared to cells treated with either drug alone. This effect was featured by the upregulated expression of Bax, cytochrome c1, p21, and cleaved caspases 8, 9, and 3, signifying the activation of both the intrinsic and extrinsic pathways of apoptosis. The sequential combination of SAHA and DAC causes a profound antitumorigenic effect in AML cell lines by inducing the expression of tumor suppressor genes.

## 1. Introduction

Besides the widely accredited genetic variation, a disrupted epigenetic outline is a well-documented hallmark of the cancer phenotype [1,2,3,4,5]. Atypical mechanisms of DNA methylation and histone acetylation, which covalently modulate chromatin architecture, play a critical role in inducing aberrant transcription of key genes regulating basic cellular processes such as cell proliferation, cell cycle regulation, and apoptosis, giving rise to a cellular growth advantage in tumor cells [1,6].

A non-permissive condensed chromatin configuration is promoted by removal of acetyl groups from lysine residues in histones, histone deacetylation, as governed by histone deacetylases (HDACs). This configuration is associated with transcriptional gene silencing. HDACs can also target nonhistone proteins implicated in regulating cellular homeostasis [7,8]. Increased activity of HDACs, their irregular recruitment to target gene-promoters, along with reduced activity of their counter parts, histone acetyl transferases (HATs) impair normally balanced acetylation in favor of hypoacetylation and intensify the tumorigenic process [6,9]. Impaired methylation patterns are, as well, a relevant feature of malignancy. Promoter CpG island hypermethylation of tumor suppressor genes, as mediated by DNA methyltransferase enzymes (DNMTs), is well-reported to associate with a closed chromatin structure and potent transcriptional silencing that inactivates key cellular pathways like DNA damage repair and apoptosis [10,11].

The two epigenetic mechanisms are functionally interdependent [12]. DNMTs with several DNA binding factors associate with HDACs to form corepressor complexes that alter chromatin architecture and, subsequently, the transcription of putative target genes [13,14]. Trichostatin A, a histone deacetylase inhibitor, induced DNA demethylation in human cancer cell lines [15], and the use of 5-azacytidine, a demethylating agent, mediated an increase in H4 acetylation [16], therefore providing evidence for an integrated crosstalk between the two epigenetic mechanisms.

Dysregulated epigenetic mechanisms are central in the pathogenesis of acute myeloid leukemia (AML), the most common type of acute leukemia in adults with poor prognosis despite induction therapy [17,18,19]. Epigenetic shifts impair the differentiation of myeloid progenitors and promote their uncontrolled clonal proliferation leading to bone marrow failure [20].

Unique DNA methylation profiles were reported in AML patient samples. Many genes were found to be differentially hypermethylated and repressed [21,22]. AML blasts also exhibited significant modifications in histone acetylation levels at more than 1000 genomic loci compared with progenitor cells with fundamental promoter regions being hypoacetylated in AML compared with progenitor cells [23].

The major role of epigenetics in myeloid leukemogenesis and the reversible nature of epigenetic alterations make epigenetic modulators attractive avenues for targeted therapy [5].

Decitabine (DAC), an azanucleoside with demethylating activity, is FDA-approved for myelodysplastic syndrome. DAC depleted DNMT1, induced DNA damage and hypomethylation, inhibited proliferation, and triggered apoptosis in AML cell lines [24,25]. HDAC inhibitors also elicited potent antitumor effects on AML at multiple levels, including cell cycle arrest induction, apoptosis, differentiation, inhibition of angiogenesis, and cell migration and invasion [26,27,28,29]. Suberoylanilide hydroxamic acid (SAHA), an HDAC inhibitor, received FDA approval for treatment of cutaneous T-cell lymphoma (CTCL) [30]. SAHA reduced cell viability, induced reactive DNA damage and apoptosis in AML cells, and enhanced the expression of numerous genes [31].

The combined use of low doses of HDAC and DNMT inhibitors may amplify their antitumorigenic effect [32]. Moreover, unfolding the molecular mechanism(s) upholding their synergistic antitumor effect would be of major scientific value. It was shown that combined DAC–SAHA treatment synergistically decreased cell proliferation, induced apoptosis, enhanced acetylation of histones, and decreased DNMT1 protein in AML cells [33]; however, the underlying mechanism(s) for these antitumor effects awaits further investigation.

The aberrant regulation of signaling pathways that control renewal and differentiation of hematopoietic stem cells (HSCs) is one of the pathogenic hallmarks and prognostic factors of human leukemias [34].

Of these pathways, the phosphoinositide 3-kinase (PI3K) AKT signaling cascade pathway displays frequent activation in AML, as shown by several studies, and is associated with decreased overall survival AML adult patients and depletion of HSCs in mice [35,36,37]. Moreover, several studies elucidated that the Wnt/β-catenin cascade, which plays an important role in normal hematopoiesis, is also aberrant in AML. In primary AML samples, elevated levels of Wnt signaling was documented with aberrant expression of β-catenin [38].

In this study, we evaluated the effect of either SAHA or DAC on viability, cell cycle regulation, and apoptosis in AML cell lines. Then, we combined SAHA and DAC together and tested the hypothesis that induced changes on cell proliferation rate and apoptotic cell death would be associated with re-regulated protein expression of the Wnt1/β catenin pathway.

## 2. Materials and Methods

### 2.1. Drugs

Suberoylanilide hydroxamic acid (SAHA) and decitabine (DAC) were purchased from Abcam (Cambridge, UK). SAHA was dissolved in dimethyl sulfoxide (DMSO) to prepare a 100 mM stock solution, whereas DAC was dissolved in sterilized distilled water to prepare a 10 mM stock solution. Both drugs were then aliquoted and frozen at −80 °C until use. Culture media was used to prepare fresh diluted drug solutions.

### 2.2. Cell Lines and Culture Conditions

KG-1 myeloblasts and U937 promonocytic leukemia cells were purchased from the American Type Culture Collection (Manassas, VA, USA) and were cultured in RPMI-1640 media supplemented with 10% fetal bovine serum (Sigma Aldrich, St Louis, MO, USA), 100 U/mL penicillin and 100 µg/mL streptomycin (Lonza, Allendale, NJ), and incubated in a humidified atmosphere with 95% air and 5% CO_2_ at 37 °C.

### 2.3. In vitro Proliferation Assays

Cell proliferation was evaluated using a WST-1 assay according to the manufacturer’s instructions. KG-1 and U937 cells were seeded in triplicate wells into 96-well cell culture plates at a density of 2 × 10^4^ cells/well and incubated overnight prior to drug treatment. Cells were then incubated with various concentrations of SAHA or DAC, alone or combined, for 24, 48, or 72 h. After incubation, 10 μL of WST-1 (Roche, Penzberg, Germany) was added to the cells and incubated for 2 h. A450 was measured using Varioskan Flash microplate ELISA plate reader (Thermo Fisher Scientific, Waltham, MA, USA). Results are expressed as proliferation percentage of treated cells relative to 100% proliferation of untreated control cells. The shown data are obtained from at least three independent experiments.

### 2.4. Cell Cycle Analysis

Cell cycle status was evaluated using propidium iodide flow cytometric analyses of cellular DNA content. KG-1 and U937 cells were seeded into 6-well cell culture plates at a density of 5 × 10^4^ cells/well and incubated overnight prior to drug treatment for 24, 48, or 72 h. Cells were then collected by centrifugation at 1500 rpm for 5 min at 4 °C, washed twice with ice-cold PBS, resuspended in 600 µL of cold PBS, fixed in 1.4 mL of cold absolute ethanol, and then stored at −80 °C for at least 12 h before staining and analysis. Fixed cell samples were then incubated with 500 µL of staining solution composed of PBS, 1 mg/mL propidium iodide (Sigma Aldrich, St Louis, MO, USA), and 100 µg/mL RNase A (Roche, Penzberg, Germany) in a volume ratio of 600:30:2 in dark for 40 min and then analyzed by flow cytometry (BD Accuri C6, Becton-Dickinson, Mansfield, MA, USA). A total of 10,000 gated events were acquired to assess the proportions of cells in different stages of the cell cycle. Analysis of cell cycle distribution was performed using C Flow Software (version 1.0.227.4, Accuri Cytometers, Ann Arbor, MI, USA).

### 2.5. Apoptosis Assay

Cell apoptosis was assessed using the Annexin V-FITC apoptosis detection kit (Abcam, Cambridge, UK) according to the manufacturer’s instructions. Cells were seeded into 6-well cell culture plates at a density of 5 × 10^4^ cells/well and incubated overnight prior to drug treatment for 24, 48, or 72 h. Cell samples were then collected by centrifugation at 1500 rpm for 5 min at 4 °C, washed twice with ice-cold PBS, resuspended in 500 µL binding buffer, stained with 5 µL Annexin V-FITC and 5 µL propidium iodide, and then incubated in dark for 5 min. Fluorescence was measured through FL-1 (530 nm) and FL-2 filters (585 nm). The early apoptotic cells (Annexin V positive only) and late apoptotic cells (Annexin V and PI positive) were then quantified.

### 2.6. Protein Extraction and Quantification

Protein extraction from cultured cells was done using Qproteome™ Mammalian Protein Prep Kit (Qiagen, Hilden, Germany) according to the manufacturer’s instructions. Cells were collected by centrifugation at 1500 rpm for 5 min at 4 °C, washed twice with ice-cold PBS, and then homogenized in lysis buffer supplemented with protease inhibitor and benzonase nuclease. Protein quantification was performed using a DC Protein Assay II kit (Bio-Rad, Germany).

### 2.7. Western Blotting

Protein samples were loaded into the wells of stacking gel and fractionated at 130 V until bromophenol blue reached the bottom of the resolving gel. Proteins were then transferred to polyvinylidene difluoride (PVDF) membranes at 0.25 A for 80 min. The blots were then blocked in 5% bovine serum albumin (BSA) prepared in PBS supplemented with 0.05% Tween-20 at room temperature for 1 h, then incubated with the primary antibody of interest prepared in 2% BSA for 1.5 h. Primary antibodies used were monoclonal antibodies for p21, cytochrome c1, Bax, caspase 3, caspase 8, caspase 9, PI3 Kinase p85 alpha (phospho Y607), AKT1 (phosphoS473), Wnt1, GSK3β and β-catenin (Abcam, Cambridge, UK), and β-actin (Santa Cruz Biotechnology, Santa Cruz, CA, USA). Secondary antibodies (Promega, USA) were used against each primary antibody. The immunoblots were visualized with enhanced chemiluminescent reagent (ECL; GE Healthcare) using ChemiDoc XRS+ imaging machine (Bio-Rad, Germany). Intensity of bands was then determined using ImageJ software (version 1.32, National Institute of Health, Bethesda, MD, USA).

### 2.8. Statistical Analysis

All experiments were performed in triplicate wells and were repeated at least three times. Results are expressed as mean ± SD. Statistical comparisons were performed using t-tests. *p* values of *p <* 0.05, *p <* 0.001, *p* < 0.0001 (*, **, *** respectively) were considered significant. GraphPad Prism software (version 5.00, GraphPad Software Inc, La Jolla, CA, USA) was used to perform statistical analyses.

## 3. Results

### 3.1. SAHA and DAC Reduce the Viability of KG-1 and U937 Cells

**Figure 1:**
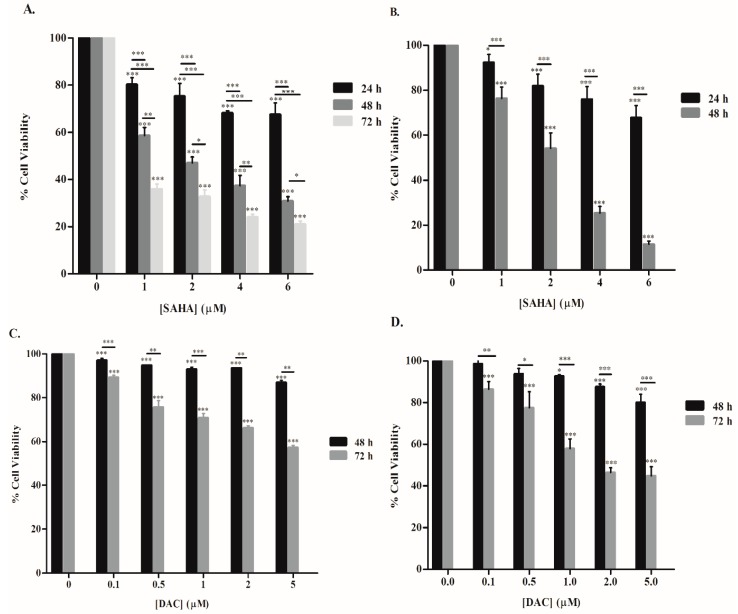
Effect of suberoylanilide hydroxamic acid (SAHA) and decitabine (DAC) on cell proliferation of KG-1 and U937 cell lines. The percentage of cell viability was calculated relative to untreated control cells using a WST-1 assay. Cell viability assays of KG-1 (**A**) and U937 (**B**) cells treated with various concentrations of SAHA (1, 2, 4, and 6 µM) for 24, 48, or 72 h. Cell viability assays of KG-1 (**C**) and U937 (**D**) cells treated with various concentrations of DAC (0.1, 0.5, 1, 2, and 5 µM) for 48 or 72 h. Data are expressed as mean ± SD of at least four independent experiments performed in triplicate. *, **, and *** indicate *p* < 0.05, *p* < 0.001, and *p* < 0.0001 respectively.

The effect of SAHA or DAC on the proliferation of KG-1 and U937 cells was measured using a WST-1 cell proliferation assay. SAHA and DAC showed a significant reduction in the proliferation of both AML cell lines in a dose- and time-dependent fashion (Figure 1). The highest assayed dose of SAHA, 6 µM, attenuated the growth of KG-1 cells by 32.5%, 69%, and 79% after 24, 48, and 72 h of treatment, respectively, with an IC50 value of 1.5 µM after 48 h of treatment (Figure 1A). A more pronounced effect was obtained by SAHA treatment on the U937 cell line, where 88.5% reduction in cell viability was attained after 48 h of treatment with an IC50 of 2.2 µM (Figure 1B). DAC, on the other hand, showed a modest, yet a significant, effect on cell viability of AML cell lines after 48 h of treatment. The 5 µM DAC dose reduced the proliferation of KG-1 and U937 cells by 13% and 20%, respectively. A more substantial effect was induced after 72 h of DAC treatment. With the 5 µM dose, the proliferation of U937 cells was lessened by 55%, with an IC50 value of 1.6 µM, and that of KG-1 cells declined by 43% (Figure 1C,D).

### 3.2. SAHA Induces Cell Cycle Arrest in the S/G2 Phase of KG-1 and U937 Cells

**Figure 2:**
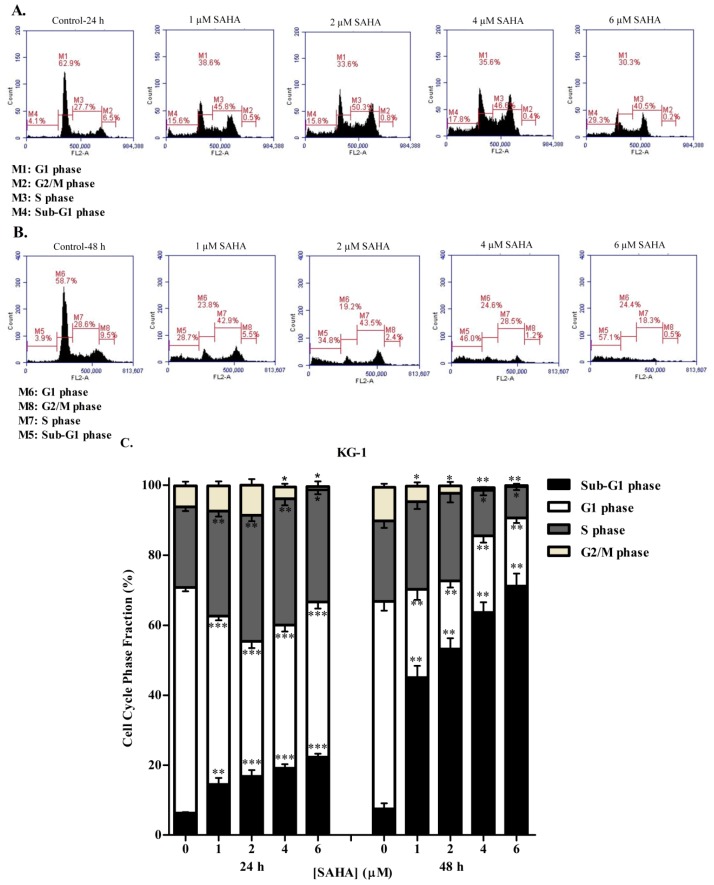
Effect of SAHA on cell cycle distribution of KG-1 cell line. Cell cycle analysis of KG-1 cells treated with SAHA (1–6 µM) for 24 h (**A**) or 48 h (**B**) respectively. Cells with < 2n DNA content were in the sub-G1 phase. Cells in G1 and G2/M phases contained 2n and 4n DNA contents respectively. Cells in between the two phases were in the S phase. The percentage of each cycle was determined using C Flow software. (**C**) Histogram analysis showing the percentage of cell cycle distribution of KG-1 cells treated with SAHA. Three separate experiments were performed for each time point and treatment condition, reported as the mean ± SD. *, **, and *** indicate *p* < 0.05, *p* < 0.001, and *p* < 0.0001 respectively.

**Figure 3:**
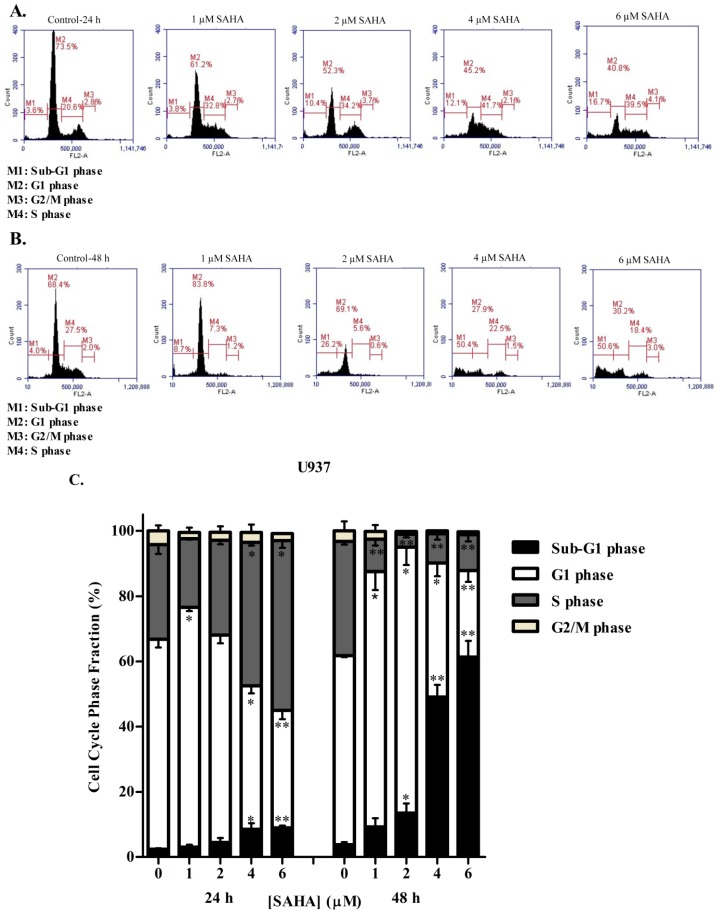
Effect of SAHA on cell cycle distribution of the U937 cell line. Cell cycle analysis of U937 cells treated with SAHA (1–6 µM) for 24 h (**A**) or 48 h (**B**) respectively. (**C**) Histogram analysis showing the percentage of cell cycle distribution of U937 cells treated with SAHA. Three separate experiments were performed for each time point and treatment condition, reported as the mean ± SD. * and ** indicate *p* < 0.05 and *p* < 0.001 respectively.

To study whether the reduction in cell growth and proliferation obtained after SAHA treatment was due to cell cycle arrest, the cell cycle status of KG-1 and U937 cells was evaluated using propidium iodide staining followed by flow cytometric analyses. The distribution of the cellular DNA content of KG-1 and U937 cells showed that, consistent with WST-1 cell proliferation assay results, SAHA induced a significant dose- and time-dependent accumulation of the cell population in the sub-G1 phase relative to control. This accumulation was accompanied by loss of cells from the G1 phase (Figure 2 and Figure 3). At 24 h, KG-1 and U937 cells treated with 6 µM SAHA significantly decreased in the G1 phase by 20% and 13%, respectively, as compared to control. This decrease was accompanied with a concomitant increase in the cell population in the S phase for both cell lines. Moreover, significant loss of cells from the G2/M phase was obtained when KG-1 cells were treated with 4 or 6 µM SAHA, whereas no significant change in the G2/M population of U937 cells was obtained (Figure 2A,C, and Figure 3A,C). This indicates that SAHA treatment of KG-1 and U937 cell lines induced an arrest in the S/G2 phase. At 48 h of SAHA treatment, however, the arrest was abolished, and the majority of the KG-1 and U937 cells were in the sub-G1 phase of dead cells (Figure 2B,C).

### 3.3. DAC Induces Cell Cycle Arrest in KG-1 and U937 Cells

**Figure 4:**
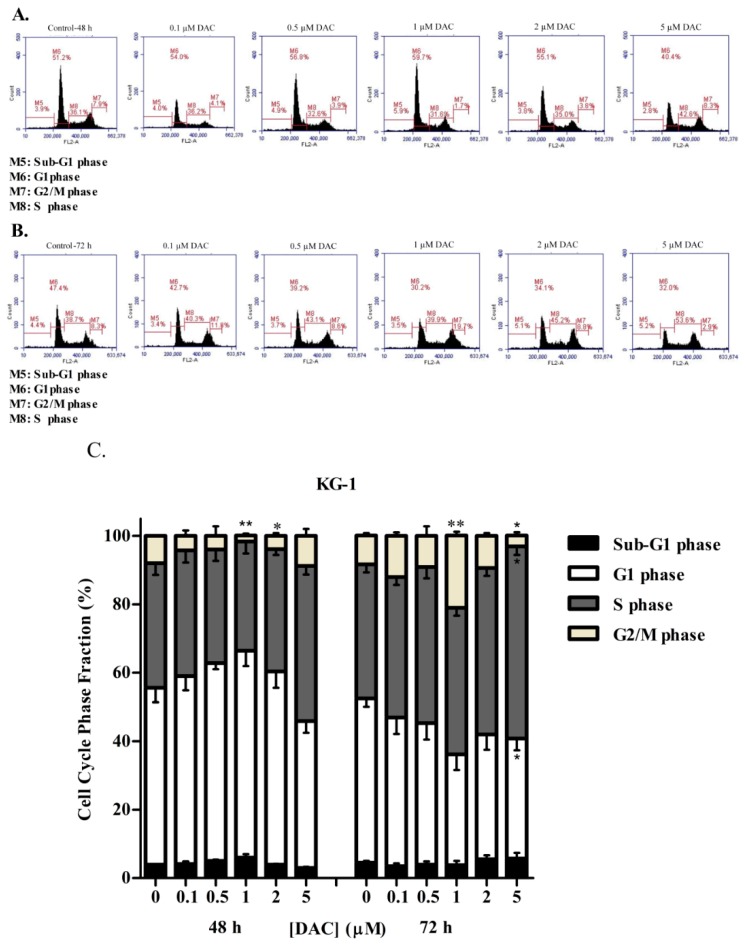
Effect of DAC on cell cycle distribution of the KG-1 cell line. Cell cycle analysis of KG-1 cells treated with DAC (0.1–5 µM) for 48 h (**A**) or 72 h (**B**) respectively. The percentage of each cycle was determined using C Flow software. (**C**) Histogram analysis showing the percentage of cell cycle distribution of KG-1 cells treated with DAC. Three separate experiments were performed for each time point and treatment condition, reported as the mean ± SD. * and ** indicate *p* < 0.05 and *p* < 0.001 respectively.

**Figure 5:**
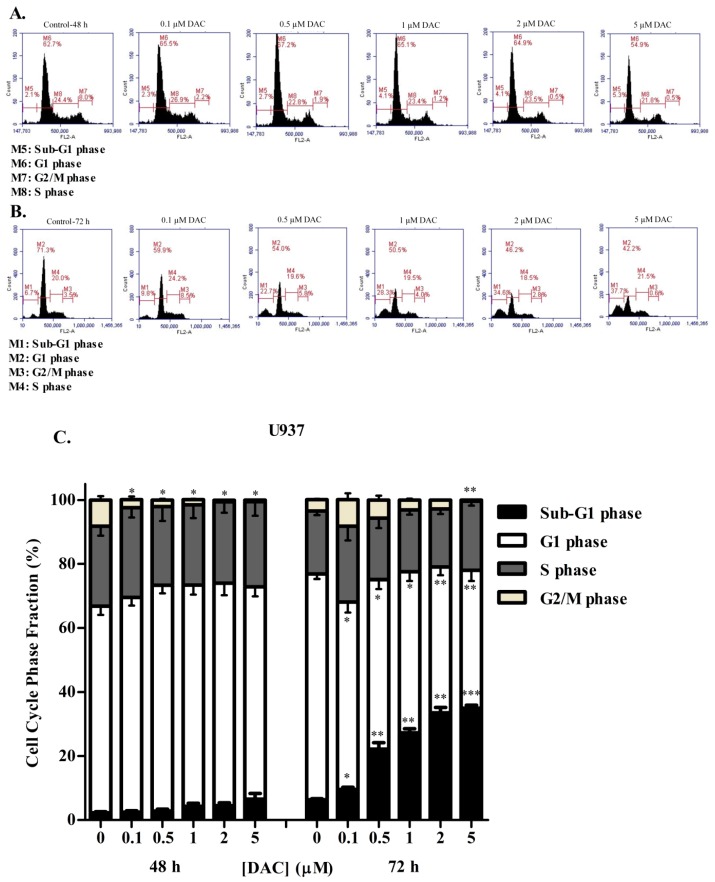
Effect of DAC on cell cycle distribution of U937 cell line. Cell cycle analysis of U937 cells treated with DAC for 48 h (**A**) or 72 h (**B**) respectively. (**C**) Histogram analysis showing the percentage of cell cycle distribution of U937 cells treated with DAC. Three separate experiments were performed for each time point and treatment condition, reported as the mean ± SD. *, **, and *** indicate *p* < 0.05, *p* < 0.001, and *p* < 0.0001 respectively.

To determine whether DAC treatment induced a similar effect to SAHA on the cell cycle distribution of KG-1 and U937 cells, the cellular DNA content was again evaluated using flow cytometry following propidium iodide staining. Our results showed that, at 48 h, low doses of DAC (0.1, 0.5, and 1 µM) caused a slight, nonsignificant increase in the KG-1 cell population at the sub-G1 and G1 phases with a concurrent loss of cells from the S and G2/M phases, therefore indicating G1 arrest (Figure 4A). At 72 h, an opposite effect was obtained, and the cells were arrested in both S and G2/M phases (Figure 4B). This reverse effect was caused by the higher doses of DAC: 2 and 5 µM. The cell population increased in S and G2/M phases relative to control samples after 48 h (Figure 4A,C). After 72 h, nevertheless, the arrest in the G2/M phase decreased in a manner, and cells increased in the sub-G1 phase instead (Figure 4B,C). U937 cells treated with DAC for 48 h showed a slight, nonsignificant increase in the cell population at the sub-G1 phase, with a significant simultaneous loss of cells from the G2/M phase relative to control sample (Figure 5A,C). After 72 h, DAC induced a dose-dependent, significant loss of U937 cells from the G1 phase and their concomitant significant buildup in the sub-G1 phase (Figure 5B,C).

### 3.4. SAHA and DAC Induce Apoptosis of KG-1 and U937 Cells

**Figure 6:**
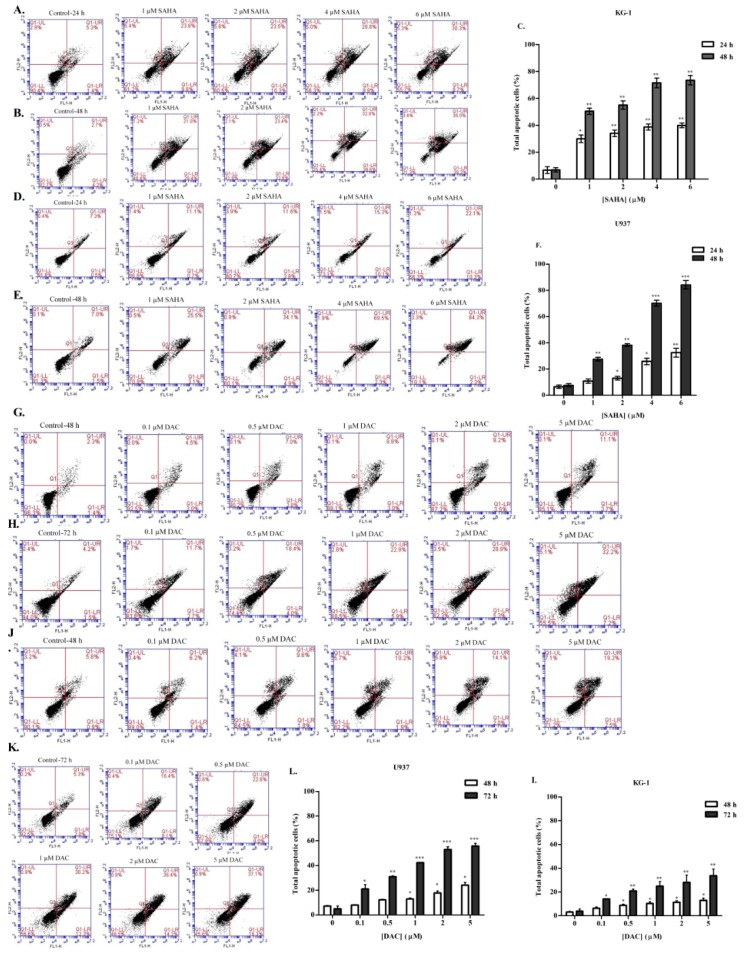
Effect of SAHA and DAC on apoptosis of KG-1 and U937 cell lines. Apoptosis of acute myeloid leukemia (AML) cells was measured by flow cytometry following double staining with Annexin V-FITC (FL1-H) and propidium iodide (FL2-H). KG-1 and U937 cells were treated with SAHA (1–6 µM) for 24 (**A** and **D**) and 48 h (**B** and **E**) respectively or with DAC (0.1–5 µM) for 48 (**G** and **J**) and 72 h (**H** and **K**) respectively. Total apoptosis was quantified following SAHA or DAC treatment in KG-1 (**C** and **I**) and U937 (**F** and **L**) respectively. Data are mean ± SD of three independent experiments. *, **, and *** indicate *p* < 0.05, *p* < 0.001, and *p* < 0.0001 respectively.

Having shown that both SAHA and DAC could induce cell cycle arrest in both KG-1 and U937 cells, we aimed then to investigate the effect of either epigenetic modulator on cellular apoptosis. For this purpose, cells were treated with SAHA or DAC for different treatment periods, stained with Annexin V-FITC and PI, and then analyzed by flow cytometry. The results showed that SAHA and DAC prompted a progressive dose-dependent increase in total apoptotic cells in both leukemia cell lines (Figure 6). Decitabine treatment of KG-1 and U937 cells for 72 h significantly augmented the population of apoptotic cells up to 33.7 ± 5.7% and 55.7 ± 2.3%, respectively, with the rate of increase in late apoptotic cells being more notable (Figure 6G,H,I,J,K,L). Similar findings were obtained with SAHA treatment: in the KG-1 cell line, all SAHA doses significantly increased the percentage of total apoptotic cells relative to untreated cells, up to 39.9 ± 1.8% and 73.5 ± 3.5% after 24 and 48 h of treatment, respectively (Figure 6A,B,C). In U937 cells, total apoptotic cells increased by 76.6 ± 3% after 48 h of treatment, with major population accumulation in the late apoptotic stage (Figure 6D,E,F).

### 3.5. Simultaneous Combination of SAHA and DAC Resulted in an Additive Cytotoxic Effect on KG-1 and U937 Cell Lines

Given that epigenetic combination treatments have been proposed to provide a more complete inhibition of cancer cell growth, and considering our findings that both SAHA and DAC elicited significant anti-cancer effect on KG-1 and U937 cell lines, we attempted to investigate their combinatorial efficacy on AML cells. In this regard, KG-1 and U937 cells were incubated simultaneously with different concentrations of DAC (0.1, 0.5, 1, and 2 µM) and the lowest assayed dose of SAHA, 1 µM, for 48 h. A WST-1 cell proliferation assay was then used to quantify cell viability.

**Figure 7:**
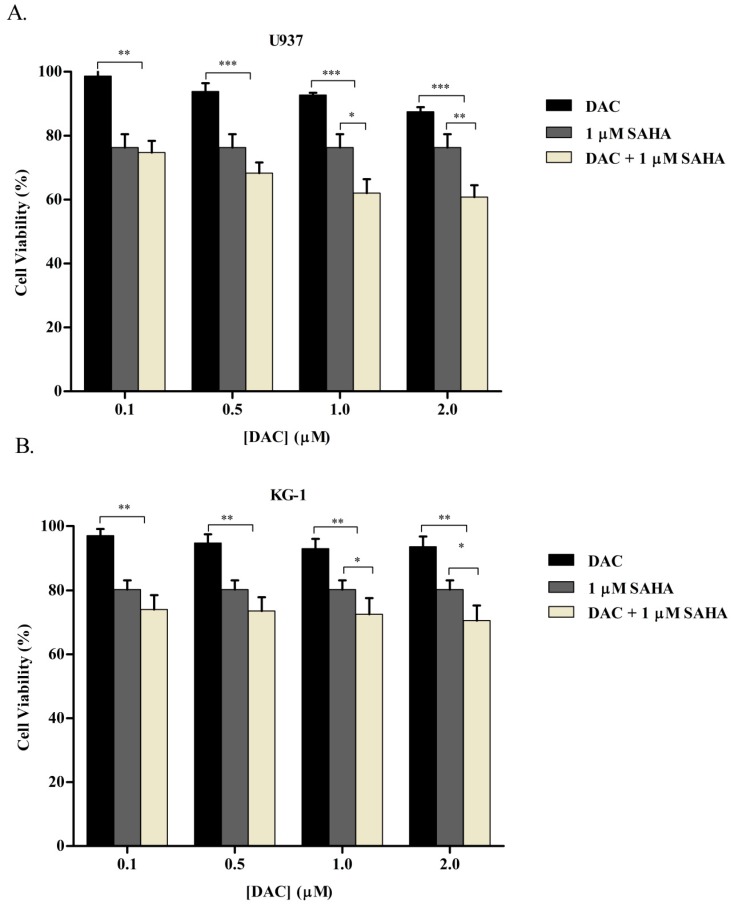
Effect of cotreatment of SAHA and DAC on cell viability of KG-1 and U937 cell lines. KG-1 (**A**) and U937 (**B**) cells were treated with 1 µM SAHA; 0.1, 0.5, 1, or 2 µM DAC; or their simultaneous combination for 48 h. Cell viability was then determined using a WST-1 assay. Data represent mean ± SD of three separate experiments. *, **, and *** indicate a significant difference between viability of cells treated with DAC or SAHA alone and cells treated with the combination of DAC and SAHA with a *p* < 0.05, *p* < 0.001, and *p* < 0.0001 respectively.

The selected doses of either SAHA or DAC caused only moderate cytotoxic cell death. As illustrated in Figure 7, the simultaneous combinatorial treatments induced an additive cytotoxic effect. Totals of 1 µM SAHA or 0.1 µM DAC reduced U937 cell proliferation by 25.1 ± 3.1% or 1.3 ± 0.2% respectively. When co-combined together, cell viability was inhibited by 26.4 ± 3.7%. The combination of SAHA with the low doses of DAC (0.1 and 0.5 µM) resulted in a nonsignificant decrease in cell viability as compared to that obtained by SAHA alone. With higher DAC doses (1 and 2 µM), although the reduction was significantly lower than the inhibition of cell viability obtained with each drug alone, the combinatorial effect was still nearly additive without any synergistic outcome (Figure 7).

### 3.6. Sequential Combination of SAHA and DAC Enhances Growth Inhibition of U937 Cell Line

**Figure 8:**
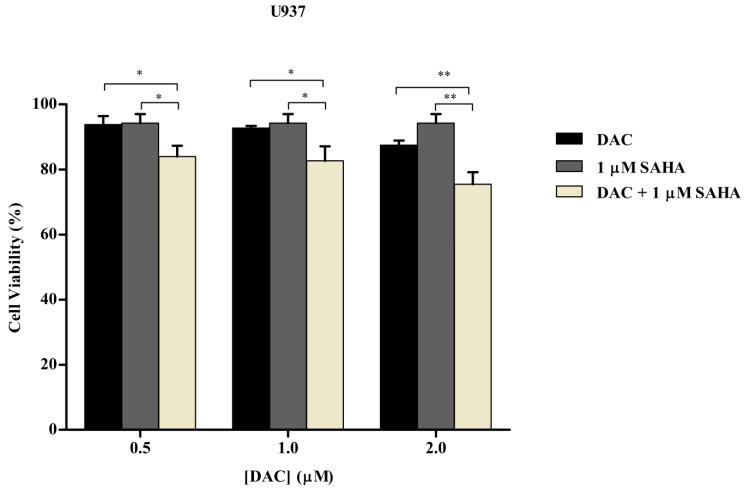
Effect of sequential combination treatment of SAHA and DAC on the inhibition of cell proliferation of the U937 cell line. Cell viability of the U937 cell line following treatment with sequential combination of DAC and SAHA for 48 h was measured using a WST-1 assay. Data represent mean ± SD of three separate experiments. * and ** indicate a significant difference in viability of cells treated with DAC or SAHA alone and cells treated with their combination, where *p* < 0.05 and *p* < 0.001 respectively.

To determine whether the antileukemic efficacy of the sequential treatment with SAHA and DAC overpowered their simultaneous administration, U937 cells were first primed with DAC (0.5, 1, or 2 µM) for 24 h, then incubated with 1 µM SAHA for an additional 24 h. After an overall period of 48 h, a WST-1 assay was used to evaluate cell viability. As depicted in Figure 8, the sequential dose combinations significantly reduced cell proliferation further than single drug treatments and achieved higher than additive cytotoxic effect. A 17.5% inhibition of cell proliferation was achieved by the sequential treatment, while 1 µM SAHA alone or 1 µM DAC alone resulted in 5.7% or 7.3%, respectively. Based on this result, the sequential combination of DAC and SAHA, rather than their simultaneous combination, was adopted to perform further experimentation.

### 3.7. The Sequential Addition of SAHA to DAC-Primed U937 Cells Expands the Apoptotic Population

**Figure 9:**
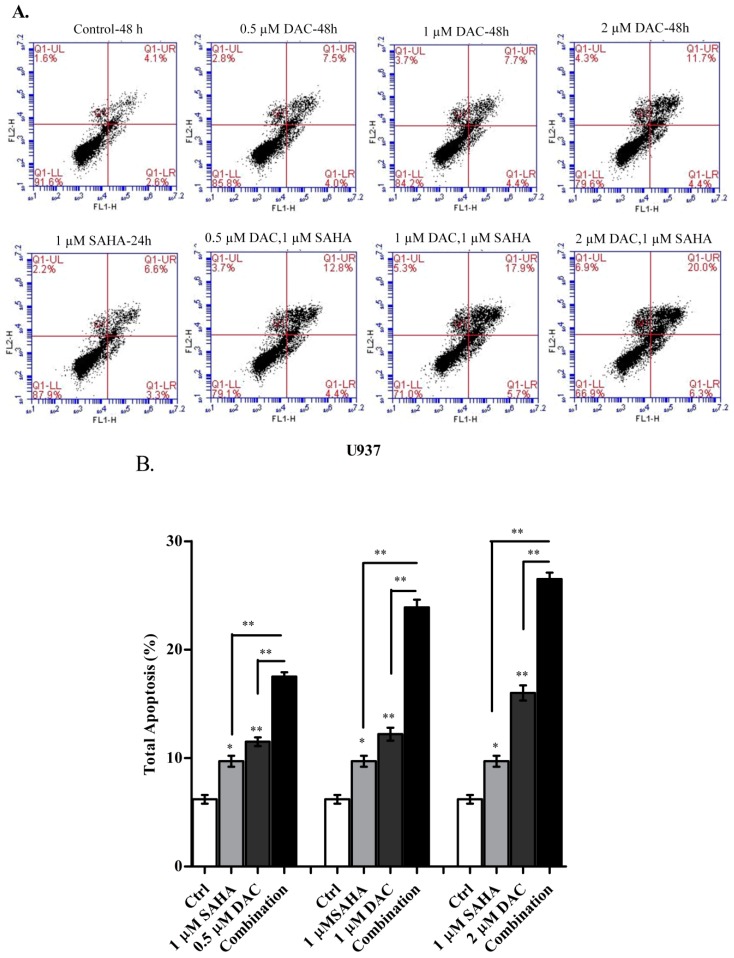
Effect of combined DAC–SAHA treatment on cell death of the U937 cell line. U937 cells were treated with DAC (0.5, 1, or 2 µM) for 48 h, 1 µM SAHA for 24 h, or first with DAC (0.5, 1, or 2 µM) for 24 h then with 1 µM SAHA for additional 24 h. Cells were then stained with Annexin V/PI and analyzed by flow cytometry. **(A**) Shows representative apoptosis profiles of U937 cells without and after treatment. (**B**) Total percentage of apoptotic cells was measured. Data represent mean ± SD of three separate experiments. * and ** indicate significantly different mean values compared with control cells and cells treated with either DAC or SAHA, where *p* < 0.05 and *p* < 0.001 respectively.

In a further attempt to determine whether the sequential combination of DAC and SAHA increased the apoptotic cell population, U937 cells were treated with DAC, SAHA, or their combination, as described before, and apoptosis was detected using Annexin V/PI double staining assay. Flow cytometry results showed that each of DAC and SAHA alone significantly increased the population of apoptotic cells ((An+/PI−) and (An+/PI+)) as compared to the control untreated cells (Figure 9B). Furthermore, the sequential treatment of U937 cells with DAC then SAHA significantly increased the population of total apoptotic cells as compared to those treated with either DAC or SAHA alone. It is worth noting that those results run in parallel to what we reported in the WST-1 cytotoxicity assays (Figure 8). These results, hence, indicated that the sequential combination improved the inhibition of cell proliferation by stimulating apoptotic cell death in the U937 cell line.

### 3.8. Sequential Treatment with DAC and SAHA Diminished the Number of Replicating Cells and Escalated the Percentage of Dead U937 Cells

**Figure 10:**
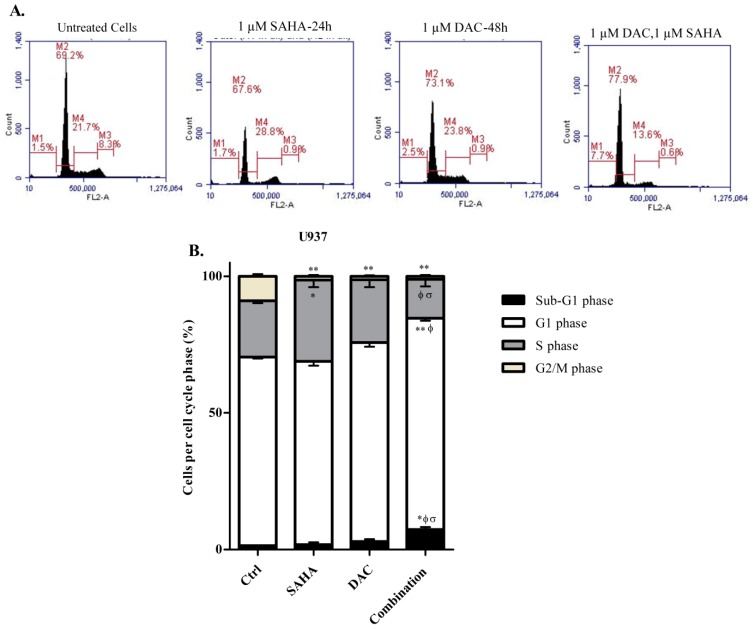
Effect of combined DAC–SAHA treatment on cell cycle of the U937 cell line. **(A**) U937 cells were treated with 1 µM DAC, 1 µM SAHA, or their combination and then stained with propidium iodide and analyzed by flow cytometry. (**B**) Histogram analysis showing the cell cycle phase distribution of U937 cells without and after treatment. Data represent mean ± SD of three separate experiments. * and ** indicate significantly different mean values compared with control untreated cells, where *p* < 0.05 and *p* < 0.001 respectively. Φ and σ indicate significantly different mean values compared with cells treated with SAHA or cells treated with DAC with *p* < 0.05.

We additionally intended to examine the effect of the sequential combination of DAC and SAHA on cell cycle phase distribution of the U937 cell line. Cells exposed to DAC, SAHA, or their sequential combination were stained with PI and examined by flow cytometry to determine the distribution of cells in the various stages of cell cycle based on their DNA content (Figure 10).

As shown previously, the treatment of U937 cells with 1 µM SAHA for 24 h alone significantly induced an S arrest and diminished the number of the cells in the G2/M phase as compared to control cells. As well, treatment with 1 µM DAC for 48 h induced a slight increase in the G1 population and also significantly reduced the G2/M population of cells as compared to control untreated cells. Cells treated with the sequential combination of DAC and SAHA elicited a significant increase in the percentage of dead cells (sub-G1 phase) as compared to control cells and cells treated with either drug alone. This increase was accompanied with a significant reduction in the percentage of the cells in the S phase (Figure 10A,B).

### 3.9. Sequential Combination of SAHA and DAC Upregulates Protein Expression of Bax, Cytochrome c1, and p21 and Increases the Cleavage of Procaspases 8, 9, and 3 in the U937 Cell Line

Attempting to decipher the molecular mechanism(s) by which the sequential combination of SAHA and DAC decreased cell viability and induced cell cycle arrest and apoptosis of AML cells, we first explored the protein expression levels of proapoptotic proteins involved in the intrinsic apoptotic pathway (caspase 9, Bax, and cytochrome c1) and the extrinsic pathway (caspase 8 and 3) and p21, a key regulator of cell cycle progression at G1 and S phases, in U937 cells, by western blot normalized to β-actin.

**Figure 11:**
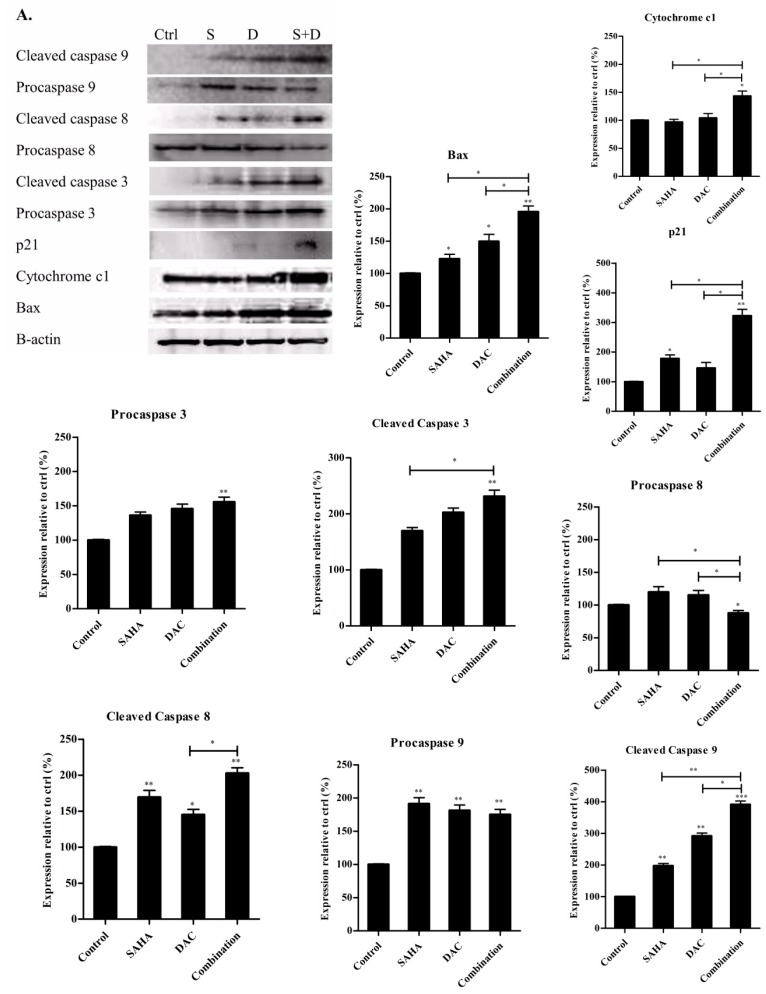
Effect of combined DAC–SAHA treatment on protein expression of Bax, cytochrome c1, p21, and caspases 8, 9, and 3 in the U937 cell line. U937 cells were treated with 1 µM SAHA for 24 h, 1 µM DAC for 48 h, or primed with 1 µM DAC for 24 h then incubated with 1 µM SAHA for additional 24 h. (**A**) Western blot for Bax, cytochrome c1, p21, procaspases 8, 9, and 3, and their cleaved and β-actin from SAHA/DAC-treated U937 cells. (**B**) Quantification analysis of the western blots. Values represent the percent expression relative to control, normalized to β-actin, for a total of 3 western blots (*n* = 3). *, **, and *** indicate significantly different mean values compared with control cells and cells treated with either DAC or SAHA, where *p* < 0.05, *p* < 0.001, and *p* < 0.0001 respectively.

Our data showed that, although treatment with either SAHA or DAC induced a modest increase in the expression levels of Bax and cytochrome c1, the combination induced a significantly greater increase when compared to control cells as well as when compared to cells treated with either drug alone. As for procaspase 9, SAHA, DAC, and their combination triggered a significant increase in the expression level relative to control cells, with combination treatment eliciting a lower increase than that induced by SAHA or DAC. The changes in the levels of procaspase 9 ran in parallel to expression levels of the corresponding cleaved product that represented a higher significant increase in the cells treated with the combination of the two epigenetic drugs (Figure 11A,B).

On the other hand, while treatment with either SAHA or DAC did not significantly change the expression of procaspase 8, the combination triggered a significant decrease relative to control cells and cells treated with either drug alone. This decrease was accompanied with a significantly higher stimulation of expression of the active cleaved caspase 8 protein in the same group of cells as well. The expression levels of an effector caspase in the two pathways, caspase 3, showed a significant increase with the combination treatment in its inactive form as well as its active cleaved product. Finally regarding p21, the treatment with SAHA or DAC separately induced a slight increase in its expression, whereas the combination induced a significant superior increase relative to control cells and cells treated with either drug alone (Figure 11A,B).

### 3.10. Sequential Combination of SAHA and DAC Upregulates Protein Expression of p-GSK3 in the U937 Cell Line

**Figure 12:**
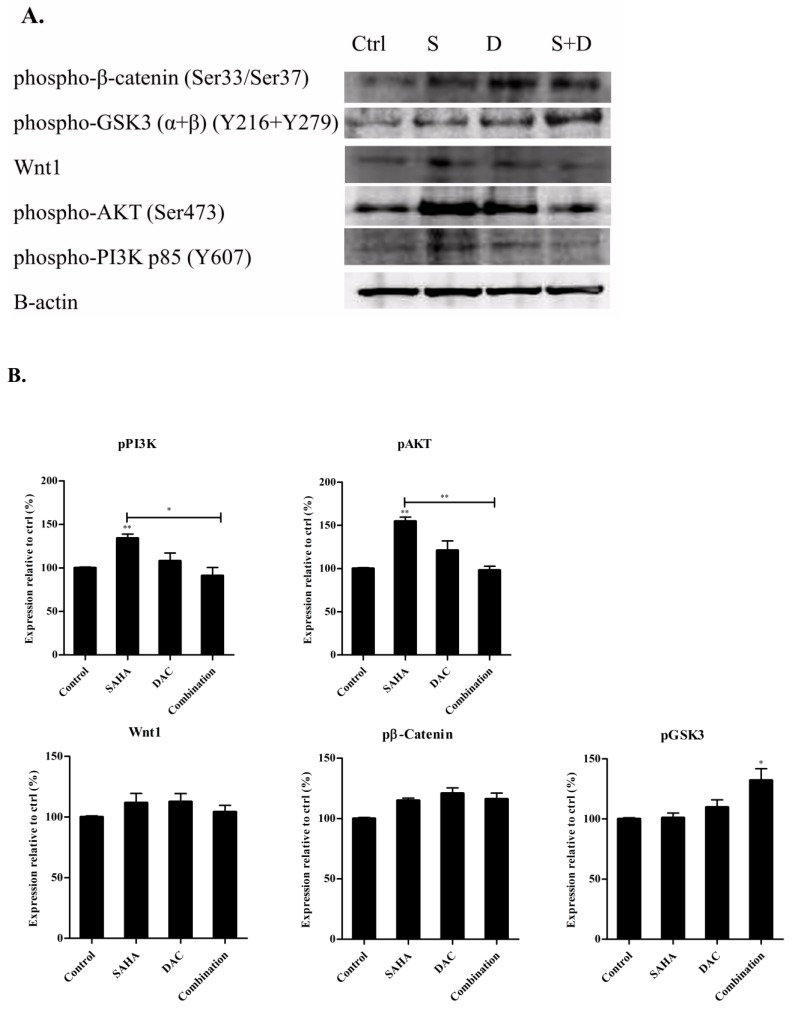
Effect of combined DAC–SAHA treatment on protein expression of p-PI3K, p-AKT, Wnt1, p-GSK3, and p-β-catenin in the U937 cell line. U937 cells were treated with 1 µM SAHA for 24 h, 1 µM DAC for 48 h, or primed with 1 µM DAC for 24 h then incubated with 1 µM SAHA for additional 24 h. (**A**) Western blot for p-PI3K, p-AKT, Wnt1, p-GSK3, p-β-catenin, and β-actin from SAHA/DAC-treated U937 cells. (**B**) Quantification analysis of the western blots. Values represent the percent expression relative to control, normalized to β-actin, for a total of 3 western blots (*n* = 3). * and ** indicate significantly different mean values compared with control cells and cells treated with either DAC or SAHA, where *p* < 0.05 and *p* < 0.001 respectively.

To further investigate whether the inhibition of cell growth and induction of apoptotic cell death of AML cells caused by combined treatment of SAHA and DAC was controlled by PI3K/AKT and Wnt/β-catenin signaling pathways, we assayed the protein expression levels of p-PI3K, p-AKT, Wnt1, p-GSK3, and p-β-catenin in U937 cells by western blot. The results showed that the combined treatment slightly downregulated the protein expression levels of p-PI3K and p-AKT relative to control expression. This decrease, however, was only significant as compared to expression levels in cells treated with SAHA alone. Moreover, the treatment of U937 cells with SAHA, DAC, or their combination did not significantly modify the expression levels of Wnt1 and p-β-catenin; nevertheless, the combinatorial treatment significantly upregulated the expression of p-GSK3 relative to control cells (Figure 12A,B).

## 4. Discussion

AML, the most common form of acute leukemias in adults, is a clonal malignant hematologic disorder characterized by impaired hematopoiesis and concurrent buildup of immature myeloid blasts [18,39]. The epigenetic characterization of AML decodes aberrant mechanisms inducing differential gene expression relevant to the malignant makeover [1,40]. Of the many ways in which epigenetic deregulations are manifested in leukemogenesis, DNA methylation and histone deacetylation are at the forefront [1,3]. Overexpressed DNMTs in AML cells induce aberrant promoter hypermethylation and concomitant gene silencing of corresponding tumor suppressors [41]. Among these, the *p15INAK4B* tumor suppressor gene is well reported to be epigenetically inactivated by irregular hypermethylation in at least half of the patients with AML [42]. On the other hand, histone hypoacetylation, as frequently induced by mutated genes affecting histone modifying enzymes, decreases chromatin accessibility and represses the transcription of tumor suppressor genes [43,44]. Mutations in the epigenetic regulators *EZH2* and *ASXL1* are found in up to 30% of AML patients [44]. The pharmacologic inhibition of DNMTs and HDACs and the enquiry of the underlying molecular mechanism of action would, therefore, hold a clinical value in targeting AML.

In this study, we first demonstrated that the hydroxamic acid HDAC inhibitor, SAHA, as well as the cytidine analogue DNMT inhibitor, DAC, attenuates the proliferation of AML cell lines KG-1 and U937, in vitro. We observed a significant time- and dose-dependent inhibition of cell growth using either epigenetic modulator. Previous reports have documented that inhibitors of HDACs and DNMTs have selective, anti-proliferative effects on multiple cancer cell lines, including AML cell lines, while maintaining normal viability and proliferation of normal blood cells [25,45,46]. The HDAC inhibitors—valproic acid, trichostatin A, and sodium butyrate—inhibited leukemic cell proliferation in a dose-dependent manner [45]. Decitabine, as well as azacitidine and zebularine, inhibited the proliferation and triggered apoptosis of AML blasts [24,25].

The anti-proliferative effect of both SAHA and DAC on KG-1 and U937 cell lines was accompanied by a significant deviation of the cell cycle status. In both cell lines, SAHA diminished the number of dividing cells and induced an arrest in the S/G2 phase. DAC, on the other hand, elicited an effect dependent on the cell line, dose, and time. In KG-1 cells, low DAC doses induced G1 or S and G2/M arrests after 48 or 72 h, respectively. The higher doses induced the S and G2/M arrests after 48 h, and the cells accumulated in the sub-G1 phase after 72 h. U937 cells showed an arrest in the G2/M phase after 48 h and also accumulated in the sub-G1 phase after 72 h of DAC treatment. The effect of SAHA and DAC in inhibiting cell cycle progression of AML cells is supported by studies that describe the role of epigenetic modulators on cell cycle dynamics by modulating the expression of cell cycle related genes [24,47,48]. SAHA was shown to modulate the expression of cyclin E and to arrest AML cells in the G2 phase [31]. As for DAC, a dual, dose-dependent mechanism of action has been reported. High doses are cytotoxic by covalently trapping the enzyme DNA methyltransferase. At lower doses, on the other hand, its antitumor effect goes back to the inhibition of DNA hypermethylation and reactivation of tumor suppressor genes [48,49]. In one previous study, DAC was shown to upregulate the expression of the cyclin-dependent kinase p21(WAF1/CIP1) and to induce G2/M cell cycle arrest in leukemia cells [47].

Besides their effect on cell cycle progression, we showed that the growth inhibitory effect of both SAHA and DAC was due to apoptosis induction in both leukemia cell lines. Our result is consistent with previous data reporting an apoptotic effect of both SAHA and DAC in AML [50,51,52]. DAC triggered DNA fragmentation, activation of caspases, and induced the expression of TNF-related apoptosis-inducing ligand (TRAIL) in AML cells [50,51]. SAHA also induced the expression of caspases 8 and 9 and upregulated the expression of death receptor 4 (DR4) and DR5 in Kasumi-1 and THP-1 leukemia cells [52].

Epigenetically based combination therapies are recently showing more promising curative results than monotherapies in several cancer types [33,53,54,55]. The maximum pharmacodynamic effect of a drug, including an HDAC or a DNMT inhibitor, will be limited by the toxicity of the provided dose. Furthermore, the epigenetic silencing occurring during tumor development has the potential to affect the sensitivity of tumor cells to chemotherapy. Phase 1 clinical trials revealed that demethylation in peripheral blood mononuclear cell (PBMCs) by decitabine is limited by the myelosuppressive activity of the drug, and the level of demethylation observed in tumors among patients was limited. Therefore, there is considerable interest in the potential use of combinatorial epigenetic therapies to overcome toxic effects of high doses of a single drug, better sensitize tumor cells to chemotherapy, overcome acquired drug resistance, as well as increase the reversal of epigenetic silencing by decitabine alone [56,57,58].

Herein we aimed to investigate the combined action of SAHA and DAC on AML cell lines. In an attempt to primarily optimize the sequence and the dose of drug administration, we first evaluated the cytotoxic efficacy of the concurrent treatment for 48 h. The doses which exhibited toxic effects without large-scale cytotoxic cell death were chosen for combination studies: 0.5, 1, and 2 μM for DAC and 1 μM for SAHA. In both KG-1 and U937 cell lines, the decrease in cell viability obtained with the combination treatments elicited an additive outcome of individual drug effects; however, no significant differences could be detected in some doses. Interestingly, U937 cells primed with DAC for 24 h, then incubated with SAHA for an additional 24 h, underwent a significant reduction in cell proliferation that surpassed the additive effect of single-agent treatments, indicating that the sequential rather than concurrent treatment is a more promising regimen for cell death induction in AML. A similar finding has been reported by Young et al., where the sequential combinations of low doses of SAHA and DAC achieved a high degree of synergy, whereas higher doses were required for the concurrent strategy to only achieve a slight synergistic effect [33]. Likewise, Yang et al. demonstrated that the sequential treatment of MOLT4 AML cells with the combination of DAC and SAHA resulted in synergistic cytotoxicity independent of the sequence used [59]. For this reason, we proceeded with the sequential regimen to identify potential mechanisms for the observed antileukemic effect in vitro.

Having shown that each of SAHA and DAC induced antileukemic effects by mechanisms that included cell cycle arrest and stimulation of apoptosis, we then examined whether the augmented decrease in cell proliferation was due to an enhancement of one or more of these mechanisms. DAC (at 0.5, 1, or 2 µM for 48 h) and SAHA (at 1 µM for 24 h), when applied individually, induced modest yet significant apoptosis in the U937 cell line. The sequential combination of those doses expanded the cell population in the late apoptotic stage (An+/PI+) representing earlier induction of apoptotic cell death. Furthermore, the enrichment of cell death obtained with the combination of DAC and SAHA was also endorsed by cell cycle analysis, which revealed a significant increase in the sub-G1 population. This is in accordance with the findings of Young et al., who reported an increase in apoptosis upon combining DAC and SAHA in other AML cell lines [33].

The antileukemic effects of our combinatorial treatment of SAHA and DAC on AML cells are featured by the upregulated expression of proapoptotic proteins Bax and cytochrome c1, as well as a remarkable induction of the cell cycle protein, p21, and significant increase in the cleavage of procaspases 8, 9, and 3, as compared to the effect obtained by SAHA or DAC treatment alone. This result explains, at least in part, the greater degree of apoptosis induction in the U937 cell line by the epigenetic combination therapy that is stimulating both the intrinsic and the extrinsic apoptotic pathways. In accordance with our results, a previous study showed that the treatment of AML cell lines, CML-T1 and HL-60, with a combination of SAHA and DAC upregulated the cleavage of procaspase 3 and 7 and induced the p53-dependent apoptotic way of cell death in CML-T1 [46].

Aiming to further decode the molecular mechanism underlying the higher anti-neoplastic effect of combined DAC and SAHA observed in our study, we postulated that the combinatorial treatment will modify the expression of Wnt/β-catenin and PI3K/AKT signaling pathways. The upregulation of these two signaling pathways is detectable in a high percentage of AML samples, and they have been implicated in the induction and progression of various cancers, including AML [35,60,61]. Our results showed that among the assayed proteins—pPI3K, pAKT, Wnt1, pGSK3, and p-βcatenin—SAHA treatment significantly upregulated pPI3K and pAKT expression, and the combination treatment significantly upregulated pGSK3 expression in U937 cells relative to control cells. This result indicates that the selected combination regimen used in the study does not act through the PI3K/AKT signaling pathway. Moreover, the increased expression of pGSK3 with no paralleled elevation in pβ-catenin expression may also reflect that GSK3 is activated by a pathway independent from the Wnt signaling pathway, such as the Ras/MAPK pathway.

## 5. Conclusions

In conclusion, this study demonstrates that the sequential combination of SAHA and DAC surpasses the concurrent combination in stimulating profound anti-tumorigenic effects in AML cells in vitro. The molecular mechanism of action includes stimulation of both the intrinsic and extrinsic apoptotic pathway. Future studies are crucial to further confirm the effectiveness of the proposed epigenetic combinatorial regime and to fully decode the underlying cellular mechanism of action.
